# Textile Supplies for Hospitals

**Published:** 1915-12-18

**Authors:** 


### TEXTILE SUPPLIES FOR HOSPITALS

### Chapter I. With Subjects Still to be Dealt With.

THE desire to do away in hospital economy with every item that can be spared and every article which can be supposed to encourage the accumulation of dust is not found inconsistent in practice with liberal purchases of articles classifiable in the broadest sense as textiles. These articles of use or wear have one connection in common. They are things woven, or prepared from fibres that are capable of being woven, and hence derive their name, although in the language of the house they may be known by some other. Major and minor, the supplies coming under this head are numerous indeed, and it does hardly any violence to terminology to include among them even the incandescent mantle upon the gas-bracket and the coir mat at the door. All that is known comprehensively as "linen"—a word that has been unjustifiably stretched to include woollens and cottons—is included. The blankets, the sheets and pillow-cases, quilts, towels, mattress-cases and most of the stuffings used inside mattresses, belong demonstrably to the textile class. The uniforms of sisters, nurses, and attendants, the dressings and some of the accessories used in surgery, belong no less to the same group, and in total these indispensable necessities of hospital work may be said to achieve an unrealised importance.

The physical necessity of these every-day requisites is beyond argument, and their economic importance needs no further illustration than to say that not very much less than one-fifth part of the large sum spent in hospital administration goes in the provision and upkeep of articles of the textile class. This two-fold importance may be conceived to justify some detailed attention to the articles themselves, their nature, origins, manufacture, supply, purchase, and treatment. Too much can hardly be known about them, and these commodities are as apt as any others to fall into an unregarding use and be accepted as descended things, a little better or a little worse than others of a similar kind. It will be practicable at least to shed some light upon their genesis and value, the peculiar properties befitting materials for particular purposes, and upon differences that are recognised to exist even if the reason of their existence is none too fully understood. In a matter so abounding in complexities, where so many qualifications enter, and where technical success is so frequently achieved only by the reconciliation of opposites, it is out of the question to reduce all knowledge to a rule of thumb. At all events, it is not beyond reasonable aspiration to convey a body of sound and useful advice and remove at least a certain number of prevalent misconceptions.

### THE SCOPE OF THE PROJECT.

With these facts in view the present series of articles is attempted, and the effort will be made to discuss the subject from all sides. Attention will be given in turn to the basis of values in textiles of every sort, with notice of the universal principles upon which values in use and in commerce are founded. The distinctive properties of the several raw materials and their combinations will be reviewed and ready means of distinguishing one fibre from another be outlined. In dealing with manufacture, the various systems and processes will be described with such particularity as will at least enable their distinctive results of different manufacturing methods to be identified. As the industrial processes are different for the separate kinds of fibre, attention will be given individually to hospital woollens, inclusive of worsteds, blankets, flannels, carpets, felts, uniform cloths and flocks. Hospital linens, including all such fine and coarse articles as are made regularly from flax, hemp, and jute, will be treated apart from the hospital cottons, which to some extent overlap or unite with them. Surgical dressings in large part are manufactured from the by-products generated in manipulating fibres for the production of cloth, and their case will be taken in due sequence.

It is hoped to give a practical bent to the whole in coupling together cause and effect and showing the reactions of methods upon materials. It is intended to reinforce the foregoing by some observations upon the methods of purchase, the quantities and qualities which may most economically be purchased, and by some consideration of the markets at large. Obviously, economy in purchasing is related intimately to economy in use and to the treatment of textiles after purchase—treatments that are sometimes more destructive than would be ideally desirable were septic conditions less to be feared. Treatments prejudicial to the life of clothes and household supplies are employed with less respectable excuse in the outer world, and it will not be professed that clothes rather than patients ought to suffer when the effects of laundering and disinfecting come to be considered. As it appears altogether probable that a list of definitions may be useful, one will be included for the assistance of those whose business lies in dealing with stores, and for their interest some standard specifications of fabrics will be given. There are concrete points respecting the quality of particular fabrics offered to them upon which hospital authorities may wish to assure themselves, and the names of institutions will be given to which reference can be made when tests would be required which are beyond the facilities of even the most fully equipped nursing establishment.

The intention being to interest and inform those who are concerned with the use of textiles in hospital work, it is hoped that no diffidence will be exhibited by readers in asking specific questions upon those points in which they are interested and would desire information. With assistance from them the series can be made of greater advantage to themselves and to others, and it can be promised in advance that no request or hint coming from them will be ignored. The British textile industries are of a magnitude difficult to conceive, with an output of goods exceeding £230,000,000 in 1907. The production engages about 1¼ million people, and the total number of persons occupied in manufacturing and dealing with textile goods in this country is not far short of three millions. The figures are mentioned in earnest of the truth that the textile business is a very great affair. In face of ramifications so vast explicit suggestions may reasonably be sought with a view to dragging into prominence those parts of an infinitely complex subject that are of most practical concern to hospital administrators. The comparative merits of different articles is one direction that the inquiries might fitly take, and there is the all-important matter of the interchange of experience between parties upon the ascertained cost of equipping and maintaining hospitals of specified sizes with their full complement of textile supplies.

### Differences, Real and Superficial.

Manifestly, there is great variety of practice within the hospitals, although scarcely so great as a tyro might rashly suppose from the bare figures published with the report of King Edward's Hospital Fund. The statement of the highest and lowest prices paid for specified articles by different authorities showed:—

| | Highest s. d. | Lowest s. d. |
|---|---|---|
| Blankets ... ... ... | 10 0 | 2 11 |
| Sheets ... ... ... | 6 11 | 2 3 |
| Towels ... ... ... | 2 6 | 0 5½ |

These disparities are differences not so much of price or value as of definition. A blanket is a blanket still, whether it be made of wool or cotton, or of mixed wool and cotton, and whether it be large or small, light or heavy. Sheets are sheets, irrespective of important variations in their sizes and of whether they are linen or cotton or coarse productions made from cotton waste. There are towels certainly worth less than 5½d., and others worth more than half a crown, and there is no final reason why one and the same institution should not, for different purposes, buy both sorts and in either event make a good bargain.

In the great world of textile industry articles are made for all purposes, economical and otherwise. Cloths are made to fit arbitrary limits of price, accommodate conventional notions of appearance, or to consult prejudices which it would be difficult to justify in a court of pure reason. It will be taken as an axiom that with all this trimming and shaping hospital requirements have absolutely nothing to do, and that what is wanted in any and every case is the indefeasibly best article for its purpose, unsophisticated to flatter the eye or the touch or to conciliate any false notions of value which may have gained currency. A general idea is abroad that the art of creating misleading appearances is a typically modern outgrowth. Actually, deceitful practices have been carried on from the earliest times, and modern knowledge of the manufacturing methods pursued in past centuries arises largely from the complaints made against the adulterators of the time. Apparently there have always been goods both sound and unsound, and it is altogether probable that there were never more of the former than there are now. If consumers fail to get goods of the quality they require, the reason is assuredly not that there are no good manufacturers.

### Important for Buyers.

A fairly intimate acquaintance with textile manufacturing is required to appreciate the degree of scruple which does exist. The care begins, of course, with the selection and rejection of raw materials for conversion first into yarn of the approved uniformity and next into cloth. The production of the woven fabric is not the end of the matter, for at that point begin the finishing operations by which the final appearance and ultimate character of the product are governed. Practices legitimate and other are followed in finishing, and around some of them custom has cast a halo, although they are not in a strict sense to the advantage of the goods. Meretriciousness should and need have no place in hospital supplies, and as every operation that is performed upon goods costs money, there are double reasons for avoiding those which subtract from the real value of the article treated. The same money spent in improving the wear or the true suitability of the stuff is laid out to irreproachable purpose, and in arranging the manufacture in this way there are the possibilities of real economy. • Buying in the ordinary market goods made to serve the ordinary purposes of lay life one may effect a bargain according to ordinary standards; but in so buying there is no guarantee of securing supplies made to meet hospital standards, and, indeed, the presumption is all to the contrary. Goods are made to the standards of the market in which they are to be sold, and for exceptional purposes one needs exceptional articles. The economy of manufacturing is very much a matter of the scale on which operations are carried on, and while it cannot be professed that manufacturers have ignored the market that the hospitals afford, the understanding between producer and consumer has been in some respects less close than it might be. It is hoped that some improved understanding may result from consideration of the problems from the dual point of view.

---

**This Series will Include:—**

The Basis of Values.

Materials and their Properties.

The Manufacturing Side.

Hospital Woollens (Blankets, Flannels, Uniforms, etc.).

Hospital Linens (Towels, Bed-ticks, etc.).

Hospital Cottons (Sheets, Towels, Calico Prints, etc.).

Surgical Dressings (Absorbent Cotton, Woven Bandages, etc.).

Methods of Purchase.

The care of Hospital Supplies.

Useful Definitions and Specifications of Standard Goods.

